# Unilateral Adrenal Hemorrhage: A Rare Complication of Anticoagulant Use

**DOI:** 10.7759/cureus.25821

**Published:** 2022-06-10

**Authors:** Batoul Nasser, Moneb S Bughrara, Hazem Alakhras, Zeinab Nasser, Omar F Jameel

**Affiliations:** 1 Oncology, Oakland University William Beaumont School of Medicine, Royal Oak, USA; 2 Internal Medicine, Oakland University William Beaumont School of Medicine, Royal Oak, USA; 3 Internal Medicine, Oakland University William Beaumont School of Medicine, Rochester, USA; 4 Internal Medicine, Henry Ford Health System, Detroit, USA; 5 Internal Medicine, Beaumont Health, Royal Oak, USA

**Keywords:** retroperitoneal hematoma, adrenal insufficiency, unilateral adrenal haemorrhage, mri images, metastatic adenocarcinoma of lung, anticoagulant therapy

## Abstract

Unilateral adrenal hemorrhage is a rare but deadly complication that can occur secondary to causes such as trauma and metastasis. A 55-year-old male with a history of metastatic lung adenocarcinoma and deep vein thrombosis managed with rivaroxaban presented with acute right abdominal and flank pain. A CT angiogram of the abdomen showed a retroperitoneal hematoma around the right adrenal gland, consistent with a unilateral adrenal hemorrhage. An MRI showed no signs of adrenal metastasis and the patient had no history of trauma. The volume of the hematoma did not change in size and the patient was hemodynamically stable, which only prompted supportive management. Anticoagulant use is a known risk factor for bilateral adrenal hemorrhage. However, this case demonstrates that unilateral adrenal hemorrhage can also be a complication, one that usually appears subclinically. It can present non-specifically but may progress to a more fatal bilateral hemorrhage. Hence, it demands a high index of suspicion for patients on systemic anticoagulation.

## Introduction

Bilateral adrenal hemorrhage is a relatively rare but potentially fatal diagnosis present in roughly 1% of postmortem examinations, with a mortality rate of approximately 15%. Its etiology can vary, but it is most commonly associated with direct anticoagulant use, heparin-induced thrombocytopenia, meningococcal septicemia (Waterhouse-Friderichsen syndrome), and autoimmune diseases such as antiphospholipid syndrome [1]. However, less common is unilateral adrenal hemorrhage which tends to be caused by primary tumors like pheochromocytoma, metastatic tumors, or direct trauma to the abdomen [2]. Metastatic infiltration can occur due to the adrenal gland’s rich vascular supply and is most frequently linked with primary lung cancer, which carries a poor prognosis [3].

Most cases of adrenal hemorrhage are found incidentally, as clinical diagnosis can be difficult. Symptoms of bilateral hemorrhage tend to be vague such as non-specific abdominal or flank pain, fever, fatigue, nausea, and vomiting [1]. When 90% or more of the cortex is affected, primary adrenal insufficiency can occur, which can present with hypotension and shock that may be refractory to fluid resuscitation. While not necessary for diagnosis, it can also present with lab findings of hypoglycemia, hyponatremia, hyperkalemia, normal anion-gap metabolic acidosis, and a rapid decline in hemoglobin due to occult bleeding. Diagnosis of unilateral adrenal hemorrhage can be even more challenging as the presentation is often subclinical, as the other gland can compensate for the loss of cortisol and aldosterone [1].

Computed tomography (CT) scan is the gold standard imaging modality for adrenal hemorrhage. Adrenal hematoma can be seen on the CT scan as a round lesion in the location of the adrenal gland and can present with periadrenal fat stranding. Magnetic resonance imaging (MRI) can be used to locate the presence of any primary or metastatic tumors [1]. Once diagnosis has been made, the patient must immediately be stabilized. Hemodynamic resuscitation begins with fluids and blood products if necessary, and can include high-dose hydrocortisone and 50% dextrose if there are symptoms of adrenal insufficiency. If the patient’s status is refractory to medical intervention, surgical exploration must be considered. An adrenal crisis can be life-threatening, therefore if the index of suspicion is high, steroids can be preemptively used to prevent progression to coma and death [1].

## Case presentation

A 55-year-old male with a history of metastatic lung adenocarcinoma and coronary artery disease presented to the emergency department with a chief complaint of acute right abdominal and flank pain. He reported associated fatigue, weakness, pain with inspiration, nausea, and vomiting, but denied fever, melena, hematuria, and history of trauma. Two years prior, he had been diagnosed with a poorly-differentiated stage IVA lung adenocarcinoma. The patient was started on osimertinib, with the goal of palliative care. Nine months prior, the patient suffered from a myocardial infarct resulting in coronary intervention with drug-eluting stent placement and was started on clopidogrel. The patient was also found to have a deep vein thrombosis (DVT) and rivaroxaban was initiated.

On examination, he was hemodynamically stable, afebrile, non-toxic appearing, with a blood pressure of 155/83 mmHg and a heart rate of 87 beats per minute. Abdominal examination demonstrated normal bowel sounds and severe right-sided abdominal tenderness on palpation. Physical exam was negative for peritoneal signs of rebound tenderness or rigidity, costovertebral angle tenderness, signs of flank bruising, and Murphy’s sign.

**Figure 1:**
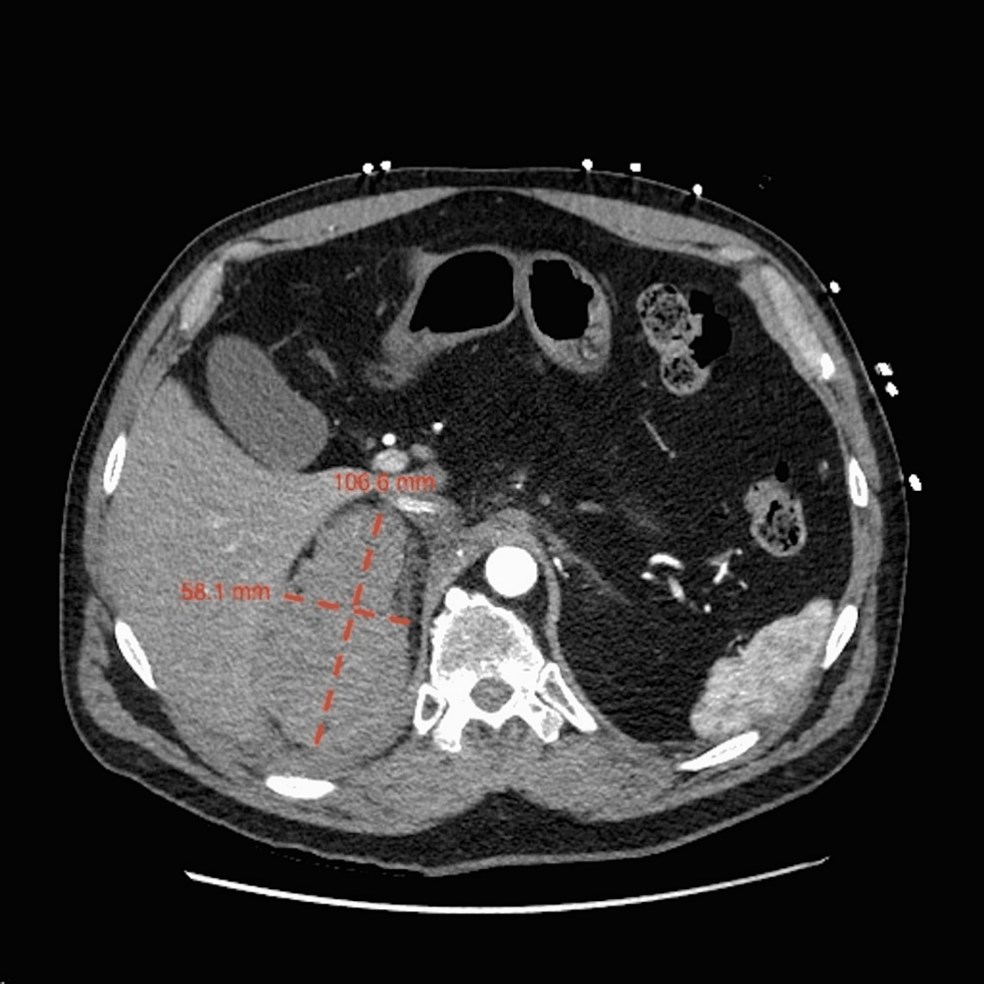
Axial CT image demonstrating a right retroperitoneal hematoma in the region of the right adrenal gland measuring 106.6 mm x 58.1 mm. There are areas of enhancement on arterial phase imaging, which represent congestion of a draining vein or delayed active extravasation.

Complete blood count (CBC) revealed a decrease in hemoglobin from a baseline of 13.6 g/dL to 10.4 g/dL, as well as evidence of leukocytosis at 12.8 bil/L. A comprehensive metabolic panel (CMP) demonstrated normal anion gap metabolic acidosis with a bicarbonate level of 15 mEq/L, but normal sodium, potassium, and glucose levels. CT angiogram (CTA) of the abdomen and pelvis showed a 106.6 mm x 58.1 mm right retroperitoneal hematoma in the region of the right adrenal gland consistent with right adrenal hemorrhage (Figure 1). An MRI of the abdomen showed no signs of adrenal metastasis.

The patient’s medications were held and steroids were not given due to no hemodynamic signs of adrenal crisis. The volume of hematoma, as well as hemoglobin levels, remained stable during the admission. This prompted no acute intervention from general surgery and interventional radiology. Due to his malignancy and high risk of thrombosis, hematology/oncology recommended resumption of his clopidogrel and rivaroxaban two weeks post-discharge.

## Discussion

While metastases are known to deposit in the adrenal gland, they rarely cause hemorrhage [4]. Regardless, due to the patient’s history of lung cancer, much consideration was given towards a metastatic infiltration of the right adrenal gland. However, CTA and MRI ruled out evidence of a metastatic deposit in adrenal glands and confirmed a unilateral adrenal hemorrhage. The variables that are most strongly associated with adrenal hemorrhage are anticoagulation therapy, thrombocytopenia, heparin use, and sepsis [5]. Since the patient was not septic, had no recent history of heparin use, and had normal platelet levels, anticoagulant use was considered as the most likely provoking factor. It is important to note that most patients with adrenal hemorrhage from anticoagulant use have normal clotting factor studies, so that cannot reliably be used as an indicator of supratherapeutic range [6].

While adrenal hemorrhage very rarely occurs in patients on anticoagulant therapy, it is implicated in about one-third of patients who present with adrenal hemorrhage [7]. Upon reviewing the literature, it is clear that most cases of adrenal hemorrhage associated with anticoagulation therapy tend to be bilateral, unlike our patient, who had a unilateral adrenal hemorrhage. One case report described a 68-year-old female with severe left upper quadrant pain, nausea, and vomiting who was diagnosed with a unilateral left adrenal hemorrhage eight days after the initiation of rivaroxaban following an uncomplicated knee arthroplasty [8]. The patient developed a right adrenal hemorrhage two days later and subsequently developed adrenal insufficiency. Another case report described a 61-year-old male with abdominal pain, nausea, vomiting, and generalized weakness who was diagnosed with a unilateral adrenal hemorrhage two weeks after the initiation of rivaroxaban following bilateral total knee replacements. He also developed adrenal insufficiency, which resolved after treatment with corticosteroids [9].

These cases highlight the importance of facilitating an earlier diagnosis in order to avoid rapid decompensation and potential progression from unilateral to bilateral adrenal hemorrhage. Given the ambiguous nature of the signs and symptoms of adrenal hemorrhage, imaging plays a key role in establishing a diagnosis, guiding management, and allowing for follow up. CT imaging is usually sufficient to establish a diagnosis as an initial imaging modality because it is time efficient and practical; however, in some cases, CT imaging is nonspecific and an MRI is warranted. While MRI is more costly and requires a patient to be stabilized, it is the most accurate imaging modality to diagnose adrenal hemorrhage, rule out the presence of a metastasis if risk factors are present, and ensure that the hematoma is not expanding [1].

There are no specific guidelines for the management of adrenal hemorrhage. In our case, conservative treatment alone was utilized and the adrenal hemorrhage resolved, which was seen in other cases of adrenal hemorrhage secondary to trauma and pregnancy [10,11]. Imaging was used to ensure that the hematoma was stable without progression, and serial labs were monitored to ensure hemoglobin levels remained stable and no signs of adrenal insufficiency developed. While adrenal insufficiency is more commonly seen in bilateral adrenal hemorrhage compared to unilateral adrenal hemorrhage, some cases of unilateral adrenal hemorrhage develop adrenal insufficiency due to the contralateral functioning adrenal gland becoming overworked [12]. If adrenal insufficiency occurs, corticosteroids need to be initiated in order to prevent death secondary to adrenal insufficiency [1]. In other cases, conservative treatment alone is insufficient; therefore, surgical exploration may be warranted. Several cases have reported angioembolization or adrenalectomy as an alternative to surgical exploration [4].

## Conclusions

This case serves to alert clinicians of rare causes and non-specific presentations of unilateral adrenal hemorrhage. It highlights the importance of having strong clinical suspicion, as morbidity and mortality can be high if diagnosis is delayed and bilateral progression occurs. If a patient presents with acute abdominal or flank pain, adrenal hemorrhage must be ruled out even if there are no obvious risk factors or signs of adrenal insufficiency. While unilateral adrenal hemorrhage is rarely associated with anticoagulant use, further research is warranted to closely study that relationship.
